# Concomitant use of anabolic androgen steroids and cabotegravir/rilpivirine leading to virological failure and development of two-class resistance

**DOI:** 10.1097/QAD.0000000000003959

**Published:** 2024-08-01

**Authors:** David Burger, Eva Wttewaal, Piter Oosterhof, Janneke Stalenhoef

**Affiliations:** a Department of Pharmacy, Radboud Institute for Medical Innovations (RIMI), Radboud UMC, Nijmegen; b Department of Internal Medicine; c Department of Clinical Pharmacy, OLVG, Amsterdam, the Netherlands.

Long-acting cabotegravir + rilpivirine (LA CAB/RPV) has demonstrated noninferiority to daily oral integrase strand transfer inhibitors (INSTI)-based combination antiretroviral therapy (ART) in multiple randomized clinical trials and real-world cohorts [1,2]. Despite their effectiveness, confirmed virological failure associated with resistance to INSTI and/or non-nucleoside reverse transcriptase inhibitor occurs [1] which may limit future treatment options. Understanding the risk factors for virological failure and resistance development with LA CAB/RPV is crucial for optimizing treatment outcomes. In this correspondence, we present a case, suggesting the concomitant use of anabolic androgen steroids as a possible cause of virological failure.

In 2013, a 27-year-old MSM was diagnosed with HIV in a country outside the Netherlands. He started with oral ART (raltegravir + emtricitabine/tenofovir disoproxil fumarate (DF)), achieving a good virological and immunological response. After moving to the Netherlands, he continued his treatment and was later switched to doravirine/lamivudine/tenofovir DF.

In March 2023, he opted to switch to LA CAB/RPV because he preferred bi-monthly injections over daily oral intake. As he initiated ART abroad and had maintained an undetectable viral load prior to starting LA CAB/RPV, we did not have access to the subtype of HIV, and analysis of baseline resistance had not been done. He began with the standard 1-month oral lead-in period. Viral load remained undetectable 3 months following the switch. However, by November 2023, his viral load increased to 635 copies/ml, confirmed 2 weeks later with a level of 814 copies/ml. Resistance testing on the latter sample identified mutations for CAB (N155S) and RPV (E138K); the HIV-1 was subtyped as A6. Plasma trough concentrations of CAB and RPV, determined by LC-MS/MS [3], were notably low: 0.91 mg/l (median population value: 1.6 mg/l; Q1 target: 1.1 mg/l [4]) and 0.026 mg/l (median population value: 0.066 mg/l; Q1 target: 0.032 mg/l [4]), respectively.

Apart from multivitamins and occasional diclofenac, his only other medications were anabolic androgen steroids (nandrolone/trenbolone) and testosterone, consistent with his activity in bodybuilding. His physique was muscular, he weighed 119.8 kg and was 1.85 m tall (BMI: 35 kg/m^2^). LA CAB/RPV injections had always been administered on the scheduled target dates. Despite his relatively high BMI, longer needles were not used as intramuscular administration was not problematic and he only had minimal subcutaneous adipose tissue. He was switched to oral boosted protease inhibitor-based ART and regained virological suppression (Table 1).

**Table 1:** Case description.

	ART	HIV viral load (copies/ml)	CAB (mg/l)	RPV (mg/l)	Testosterone (nmol/l)
27-3-2023	Oral lead-in CAB/RPV				
30-3-2023		<30			
25-4-2023	Injection CAB/RPV				
26-5-2023	Injection CAB/RPV				
26-6-2023		<30			
28-7-2023	Injection CAB/RPV				
26-9-2023	Injection CAB/RPV				
16-11-2023		635			
29-11-2023		814	0.91	0.026	1.1^a^
6-12-2023	Oral DRV/c/FTC/TAF				
25-1-2024		33			
10-5-2024		<30			

ART, antiretroviral therapy; CAB, cabotegravir; DRV, darunavir, c, cobicistat; FTC, emtricitabine; RPV, rilpivirine; TAF, tenofovir alafenamide fumarate.

a LH 0,2 IU/l; FSH 0,5 IU/l; SHBG 22 nmol/l.

We hypothesize that in this person with HIV and the unfavorable A6 subtype, the concomitant use of anabolic androgen steroids, along with testosterone, may have increased the clearance of CAB and RPV, leading to low trough levels, inadequate virological suppression and the development of two-class resistance. Several lines of evidence support this hypothesis.

First, anabolic androgen steroids mimic the effects of testosterone, which can influence hepatic metabolic enzymes such as CYP3A (relevant for RPV) and/or UGT1A1 (relevant for CAB) [5,6]. For instance, Walle *et al.*[7] demonstrated in a study with healthy volunteers that propranolol glucuronidation was positively correlated with testosterone levels, either endogenous or supplemented. Second, the combination of anabolic androgen steroids and intensive physical activity may increase blood flow at the site of injection of the LA ART, leading to an increased release from the depot and subsequent increased elimination of both agents. Although this second explanation may be an indirect mechanism, its impact on drug exposure is similar. In support of this, Thoueille *et al.*[8] reported faster elimination of both CAB and RPV on LA CAB/RPV every 2 months in a young athletic person injecting anabolic steroids, confirming our observation.

To date, most attention has focused on baseline risk factors for predicting virological failure on LA CAB/RPV. These include proviral RPV RAMs, HIV-1 subtype A6/A1, and a BMI at least 30 kg/m^2^. Only individuals with at least two of these risk factors have a statistically significant increased risk for virological failure [9,10]. We were unaware that our person with HIV had subtype A6 or had proviral RPV RAMS as his viral load had consistently been undetectable. This raises the question of whether initiating LA CAB/RPV is advisable for persons with HIV having an unknown virus subtype at treatment initiation, even if the likelihood of this mutation is low. Although a BMI at least 30 kg/m^2^ is also a risk factor for virological failure, we do not believe this was relevant in our case as he was not obese and intramuscular injection was not problematic.

In the absence of further evidence regarding the impact of anabolic androgen steroids on CAB + RPV metabolism, we recommend implementing therapeutic drug monitoring and/or intensified viral load monitoring to further prevent virological failure and resistance development in these people with HIV.

## Acknowledgements

### Conflicts of interest

There are no conflicts of interest.

## References

[1] Overton ET Richmond G Rizzardini G Thalme A Girard PM Wong A. Long-acting cabotegravir and rilpivirine dosed every 2 months in adults with human immunodeficiency virus 1 type 1 infection: 152-week results from ATLAS-2 M, a randomized, open-label, phase 3b, noninferiority study. *Clin Infect Dis* 2023; 76:1646–1654. 36660819 10.1093/cid/ciad020 PMC10156123

[2] Thoueille P Saldanha SA Schaller F Choong E Munting A Cavassini M. Swiss HIV Cohort Study. Real-world trough concentrations and effectiveness of long-acting cabotegravir and rilpivirine: a multicenter prospective observational study in Switzerland. *Lancet Reg Health Eur* 2024; 36:100793. 38162253 10.1016/j.lanepe.2023.100793 PMC10757247

[3] Bevers LAH van Ewijk-Beneken Kolmer EWJ Te Brake HML Burger DM. Development, validation and clinical implementation of a UPLC-MS/MS bioanalytical method for simultaneous quantification of cabotegravir and rilpivirine E-isomer in human plasma. *J Pharm Biomed Anal* 2024; 238:115832. 37976991 10.1016/j.jpba.2023.115832

[4] ANRS-MIE-AC43 Pharmacologic and Resistance groups: recommendations for therapeutic drug monitoring of CABOTEGRAVIR and RILPIVIRINE during long-acting injectable administration of Vocabria/Rekambys every 2 months in HIV-infected patients. Available at: https://sfpt-fr.org/images/documents/STP/Guidelines_TDM_Cabotegravir_Rilpivirine_Long-acting_CARLA_ANRS_AC43_v1_131021_englishversion.pdf. [Accessed 26 March 2024]

[5] Cirrincione LR Huang KJ. Sex and gender differences in clinical pharmacology: implications for transgender medicine. *Clin Pharmacol Ther* 2021; 110:897–908. 33763856 10.1002/cpt.2234 PMC8518665

[6] Le A Huang KJ Cirrincione LR. Regulation of drug-metabolizing enzymes by sex-related hormones: clinical implications for transgender medicine. *Trends Pharmacol Sci* 2022; 43:582–592. 35487786 10.1016/j.tips.2022.03.006

[7] Walle T Walle K Mathur RS Palesch YY Conradi EC. Propranolol metabolism in normal subjects: association with sex steroid hormones. *Clin Pharmacol Ther* 1994; 56:127–132. 8062488 10.1038/clpt.1994.115

[8] Thoueille P Alves Saldanha S Schaller F Munting A Cavassini M Braun D. Real-life therapeutic concentration monitoring of long-acting cabotegravir and rilpivirine: preliminary results of an ongoing prospective observational study in Switzerland. *Pharmaceutics* 2022; 14:1588. 36015214 10.3390/pharmaceutics14081588 PMC9413113

[9] Cutrell AG Schapiro JM Perno CF Kuritzkes DR Quercia R Patel P. Exploring predictors of HIV-1 virologic failure to long-acting cabotegravir and rilpivirine: a multivariable analysis. *AIDS* 2021; 35:1333–1342. 33730748 10.1097/QAD.0000000000002883 PMC8270504

[10] Orkin C Schapiro JM Perno CF Kuritzkes DR Patel P DeMoor R. Expanded multivariable models to assist patient selection for long-acting cabotegravir + rilpivirine treatment: clinical utility of a combination of patient, drug concentration, and viral factors associated with virologic failure. *Clin Infect Dis* 2023; 77:1423–1431. 37340869 10.1093/cid/ciad370 PMC10654860

